# Sensory Neurons Do Not Induce Motor Neuron Loss in a Human Stem Cell Model of Spinal Muscular Atrophy

**DOI:** 10.1371/journal.pone.0103112

**Published:** 2014-07-23

**Authors:** Andrew J. Schwab, Allison D. Ebert

**Affiliations:** Department of Cell Biology, Neurobiology and Anatomy, Medical College of Wisconsin, Milwaukee, Wisconsin, United States of America; University of Edinburgh, United Kingdom

## Abstract

Spinal muscular atrophy (SMA) is an autosomal recessive disorder leading to paralysis and early death due to reduced SMN protein. It is unclear why there is such a profound motor neuron loss, but recent evidence from fly and mouse studies indicate that cells comprising the whole sensory-motor circuit may contribute to motor neuron dysfunction and loss. Here, we used induced pluripotent stem cells derived from SMA patients to test whether sensory neurons directly contribute to motor neuron loss. We generated sensory neurons from SMA induced pluripotent stem cells and found no difference in neuron generation or survival, although there was a reduced calcium response to depolarizing stimuli. Using co-culture of SMA induced pluripotent stem cell derived sensory neurons with control induced pluripotent stem cell derived motor neurons, we found no significant reduction in motor neuron number or glutamate transporter boutons on motor neuron cell bodies or neurites. We conclude that SMA sensory neurons do not overtly contribute to motor neuron loss in this human stem cell system.

## Introduction

Spinal muscular atrophy (SMA) is an autosomal recessive disorder that leads to muscle weakness, respiratory distress, paralysis, and early death due at least in part to loss of motor neurons in the spinal cord. SMA is most often caused by deletion of the survival motor neuron (*SMN*) gene. Humans have two copies of *SMN*: a telomeric copy (*SMN1*) and a centromeric copy (*SMN2*) [1]. *SMN1* produces a full-length protein found in both the cytoplasm and the nucleus and is involved in biogenesis of RNA proteins, RNA transcription, and pre-mRNA splicing [2]–[4]. In SMA, total full-length SMN protein is drastically reduced due to the absence of *SMN1*, and *SMN2* is not able to fully compensate due to generation of an alternative spliced protein (SMNΔ7) [5]–[8].

Although SMN is lost in all cell types, it remains to be fully understood why motor neurons are particularly vulnerable. Recent studies have suggested that SMN is important in U12-dependent splicing events necessary for proper motor neuron function [9], but evidence also suggests that other cell types are affected, including astrocytes, sensory neurons, Schwann cells, and skeletal muscle that may each contribute to or exacerbate motor neuron loss [10]–[18]. In this regard, we have shown that motor neurons generated from SMA patient derived induced pluripotent stem cells (iPSCs) show significant loss through an apoptotic process by 6 weeks in culture [19], [20]. Moreover, we recently showed that astrocytes derived from SMA iPSCs are activated and exhibit abnormal calcium homeostasis and reduced growth factor production prior to the overt motor neuron loss [16]. Other studies using human patients and nerve biopsies have shown reduced nerve conduction velocity and inexcitability of sensory neurons [21]–[23]. Similarly, there is evidence from a mouse model of SMA that dorsal root ganglia (DRG) sensory neurons have reduced neurite outgrowth compared to control [11] and spinal afferent synaptic connections onto the motor neurons are reduced before the onset of significant motor neuron loss [12], [14]. Loss of the proprioceptive sensory neurons and primary afferent boutons was more severe for synapses formed on motor neurons projecting to proximal muscles [12], [14], which is consistent with the pronounced atrophy observed in the limbs, but the significance of these data is debated as other studies demonstrated that sensory neuron deficits in SMA were a consequence of motor neuron dysfunction, rather than a cause [10], [13], [24], [25]. Data from Drosophila have shown that SMN replacement is necessary in sensory neurons and interneurons to restore motor neuron and muscle function [15]. Finally, very recent studies have found that mRNAs related to synaptic formation and sensory-motor circuitry are dysregulated in SMA mice spinal cord prior to motor neuron loss [26]. Decades of research confirm that direct and indirect sensory afferent innervations onto spinal cord motor neurons, such as in the spinal reflex circuit, are critical to motor neuron function and subsequent motor output [27]. Synaptic activity is critical for neuronal survival through inhibition of apoptotic cascades [28]–[30], and significant evidence exists suggesting that SMA motor neurons die through an apoptotic mechanism [20], [31]–[33]. Taken together, these data suggest that non-motor neuron cell types may actively contribute to the SMA disease phenotype by directly affecting motor neuron survival, but further investigation is needed.

Traditional in vitro and in vivo animal models are readily available to study neurodegenerative diseases, and the groundbreaking iPSC technology has opened up additional avenues of exploration into human development and disease [34]–[36]. Here we generated sensory neurons from SMA and control iPSCs in an effort to determine if SMA iPSC-derived sensory neurons directly contribute to motor neuron loss. We followed an established sensory neuron differentiation protocol [37] and show that SMA and control iPSCs generate similar numbers of peripherin positive sensory neurons, with small populations of these neurons being nociceptors and proprioceptors. Using direct co-culture methods, we asked whether SMA iPSC-derived sensory neurons caused loss of healthy iPSC-derived motor neurons indicating a direct role of sensory neurons in SMA disease progression. Neither total motor neuron number nor vesicular glutamate transporter staining on healthy iPSC-derived motor neurons was significantly reduced when cultured with SMA iPSC-derived sensory neurons. Therefore, we conclude that interactions with SMA iPSC-derived sensory neurons are not sufficient to induce motor neuron loss in a human iPSC system.

## Materials and Methods

### Cell culture

Two independent control iPSC lines (4.2 and 21.5) and two independent SMA iPSC lines (3.6 and 7.12) were used [19], [20], [38]. iPSCs were grown in feeder free conditions on Matrigel substrate (BD Biosciences) in Nutristem medium (Stemgent). Neural stem cells (EZ Spheres) were generated by lifting intact colonies following dispase treatment (1 mg/ml, Gibco) and placing them directly into a human neural progenitor growth medium (Stemline, Sigma) supplemented with 100 ng/ml basic fibroblast growth factor (FGF-2, Millipore), 100 ng/ml epidermal growth factor (EGF, Miltenyi Biotec), and 5 µg/ml heparin (Sigma) in ultra-low attachment flasks and were passaged weekly using a chopping technique as previously described [39].

### Sensory neuron differentiation

Induction of sensory neurons was accomplished using an established protocol [37]. Briefly, human iPSC colonies were grown to confluence on poly-ornithine/Matrigel coated coverslips. Once 80–90% confluence was reached, the Nutristem medium was replaced with knockout serum replacer (KSR) medium supplemented with 1 µM dorsomorphin (Tocris) and 10 µM SB431542 (Stemgent) on days 0–5. KSR medium was prepared by supplementing 820 ml of Knockout DMEM, with 150 ml knockout serum replacement, 1 mM L-glutamine, 100 µM MEM nonessential amino acids, 1% antibiotic-antimycotic (anti-anti) (all from Life Technologies), and 0.1 mM β-mercaptoethanol (Sigma). Cells were fed daily and N2 medium (1∶1 DMEM (Sigma)/F12, 2% N2, 1% anti-anti; Life Technologies) was added in 25% increments every other day starting on day 4 (100% N2 on day 10). 3 µm CHIR99021 (Stemgent), 10 µm SU5402 (Santa Cruz Biotechnology), and 10 µm DAPT (Tocris) were added on days 2–10 to induce sensory neurons. After day 10, long term medium consisted of N2 medium supplemented with brain derived neurotrophic factor (BDNF; 25 ng/ml; Peprotech), glial cell line derived neurotrophic factor (GDNF; 25 ng/ml; Creative Biomart), nerve growth factor (NGF; 25 ng/ml; R&D Systems), ascorbic acid (200 µg/ml; Sigma), and cAMP (1 µM; Sigma). Long term maintenance medium was replaced every 2 days.

### Calcium imaging

iPSC-derived sensory neuron cultures were cultured for 2 weeks and functionally tested using ratiometric live-cell calcium imaging using dual-wavelength fluorescent calcium indicator FURA-2AM (Invitrogen) to detect intracellular calcium levels. Cells were loaded with 2.5 µL/ml FURA-2AM in 2% BSA (Sigma) for 1 hour at room temperature, washed in extracellular buffer for 20 minutes, and mounted onto a perfusion chamber and superfused with extracellular buffer at 6 mL/min. Coverslips were superfused with extracellular buffer containing 50 mM KCl for 30 secs. Fluorescence images were taken with a cooled CCD camera (CoolSNAP FX; Photometrics, Tucson, AZ) before and after stimulation. Metafluor imaging software was used to detect and analyze intracellular calcium changes throughout the experiment (Molecular Devices, Sunnyvale, CA) where a ≥20% increase in intracellular calcium from baseline constituted a response. Extracellular buffer contained 150 mM NaCl, 10 mM HEPES, 8 mM glucose, 5.6 mM KCl, 1 mM MgCl_2_, and 2 mM CaCl_2_ (all from Sigma).

### Motor neuron differentiation/co-culture

Motor neuron differentiation from the EZ spheres was performed as previously described [19], [39]. Briefly, the neural progenitor growth medium was replaced with neural induction medium (1∶1 DMEM/F12, 1% N2, 1% NEAA, heparin (1 mg/ml), 1% anti-anti) supplemented with retinoic acid (0.1 µM; Sigma) for one week followed by supplementation with retinoic acid (0.1 µM) and purmorphamine (1 µM; Stemgent) for an additional week changing half the medium every 3–4 days. After two weeks of differentiation, the spheres were dissociated with accutase (Millipore) and plated onto 2 week old sensory neuron cultures at a density of 30,000 cells per coverslip. Long term medium consisted of N2 medium supplemented with BDNF (25 ng/ml), GDNF (25 ng/ml), NGF (25 ng/ml), ascorbic acid (200 µg/ml), cAMP (1 µM). The medium was replaced every 2 days.

### Immunocytochemistry

Plated cells were fixed in 4% paraformaldehyde (PFA; Fisher Scientific) for 20 minutes at room temperature and rinsed with PBS. Nonspecific labeling was blocked and the cells permeabilized with 5% normal goat serum (Millipore) and/or 5% normal donkey serum (Millipore) and 0.2% Triton X-100 (Sigma) in PBS for 30 minutes at room temperature. Cells were rinsed with PBS and then incubated with primary antibodies for one hour at room temperature or overnight at 4°C. Cells were subsequently labeled with the appropriate fluorescently-tagged secondary antibodies. Hoechst nuclear dye was used to label nuclei. Primary antibodies used were rabbit anti-Peripherin (Millipore, AB1530), mouse anti-βIII Tubulin (Tuj1, Promega, G7121), rabbit anti-NTRK1 (Millipore, 06-574), rabbit anti-TRPV1 (Novus Biologicals, NBP1-97417), mouse anti-GFAP (Cell Signaling, 3670), rabbit anti-Parvalbumin (Calbiochem, PC255L), mouse anti SMI32R (Covance, SMI-32R), guinea pig anti-VGlut1 (Millipore, AB5905), and myelin protein zero (ProteinTech, 10572-1-AP). Secondary antibodies included donkey anti-mouse AF488 (Invitrogen, A21202), goat anti-rabbit RhoRed (Invitrogen, R6394), and goat anti-guinea pig AF488 (Life Technologies, A11073).

### Imaging and quantification

At least five images were taken on each of at least three different fluorescently labeled coverslips per timepoint using a Nikon inverted microscope and Spot imaging software. All experiments were repeated at least 2 times. The images were analyzed for antigen specificity using MetaMorph Software (Molecular Devices Inc.). Data were statistically analyzed via one-way ANOVA or Student’s t-test as appropriate with Prism software, α = 0.05 (GraphPad). Data are presented as the average ± S.E.M. using two independent control lines and two independent SMA lines.

### Ethics statement

The privacy of the fibroblast cell sources from which the iPSCs were derived is maintained by Coriell Institute for Medical Research. The use of iPSCs has been approved by the Stem Cell Research Oversight Committee at the Medical College of Wisconsin.

## Results and Discussion

### Sensory differentiation and characterization from SMA and control iPSCs

**Figure 1:**
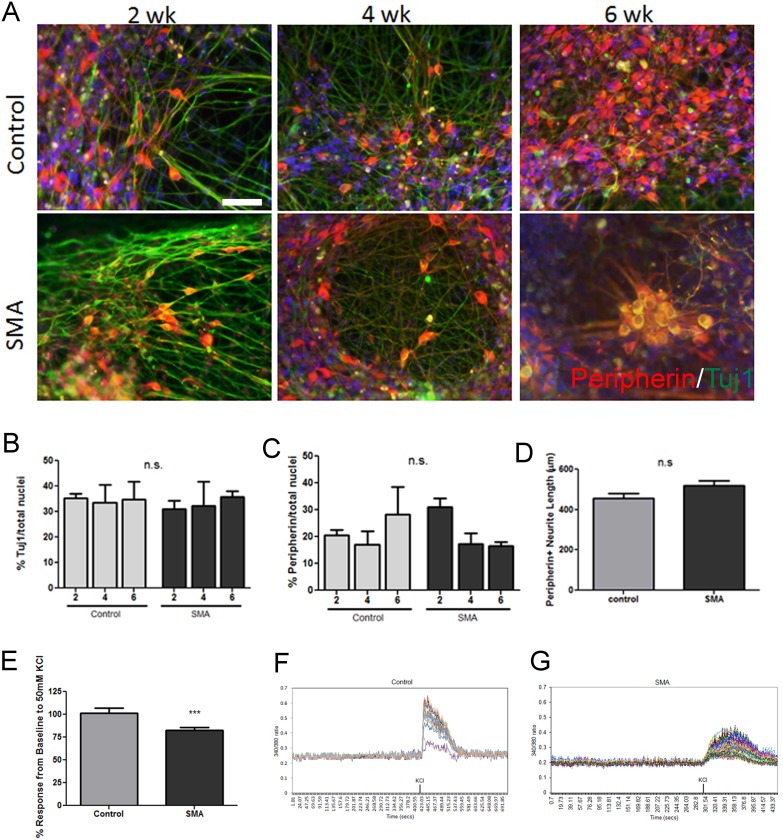
Consistent generation of sensory neurons from control and SMA iPSCs. (A) Control and SMA iPSCs acquire a Tuj1+ (green)/peripherin+ (red) sensory neuron phenotype by 2 weeks of differentiation that remained consistent through 6 weeks of differentiation. Nuclei are labeled with Hoechst nuclear dye (blue). Quantification of the Tuj1+ population (B) and peripherin+ population (C) show no significant difference in neuronal differentiation efficiency between control and SMA iPSCs at any time point. (D) At 6 weeks of differentiation there was no difference in neurite length between control and SMA iPSC-derived sensory neurons. (E) Ratiometric live cell calcium imaging showed a significant reduction in calcium response to KCl depolarization in the SMA iPSC-derived sensory neurons at 2 weeks of differentiation compared to controls. Representative imaging traces of at least 30 individual cells are shown for (F) one control iPSC line and (G) one SMA iPSC line. KCl indicates the time at which the depolarizing stimulus was added to the cultures. n.s. = not significant by ANOVA (B and C) or by Student’s t-test (D). ***p = 0.0008 by Student’s t-test (E). Scale bar = 50 µm.

To examine the functional properties of SMA sensory neurons, we utilized human induced pluripotent stem cells (iPSCs) derived from SMA patients and healthy individuals [19], [20], [38]. Control and SMA iPSCs were cultured as adherent monolayers for sensory neuron induction following a previously established protocol [37]. This protocol allows for the efficient generation of peripherin positive sensory neurons within 2 weeks of differentiation. Peripherin labels thin, unmyelinated sensory neurons, which includes both nociceptive and proprioceptive neurons. The cellular compositions derived from the differentiations were analyzed by immunostaining with specific cellular markers at 2, 4, and 6 weeks of culture. Importantly, both control and SMA iPSCs acquired a sensory neuronal phenotype as identified by βIII tubulin (Tuj1) and peripherin (Figure 1A). Quantification of Tuj1 and peripherin positive neurons resulted in approximately 35% Tuj1+ and 20% peripherin+ cells over the course of the differentiation period from both control and SMA iPSCs (Figure 1B, C). Previous studies have found decreased neurite extension from isolated DRG sensory neurons from SMA mice [11] as well as axon degeneration from the sural nerve in human SMA patients [23]. However, neither of these characteristics was observed in human SMA iPSC-derived sensory neurons (Figure 1A, D). Interestingly, we used ratiometric live cell calcium imaging to test functional responses to KCl depolarizing stimulation and found a significant reduction in the intracellular calcium response in SMA iPSC-derived sensory neurons compared to control iPSC-derived sensory neurons (Figure 1E–G), which agrees with data collected from one SMA patient indicating a reduced neuronal excitability [22]. The reason for some of the discrepancies between data presented here and previous reports [11], [23] is unknown. The sensory neuron differentiation protocol generates neurons similar to DRG neurons [37], which facilitates comparisons with the mouse study [11], but species differences and/or culture conditions may contribute to different results. Additionally, the sural nerve studied by Rudnik-Schoneborn and colleagues [23], which is a sensory nerve found in the leg that innervates the foot may be too distinct from DRG-like neurons to allow direct comparisons.

**Figure 2:**
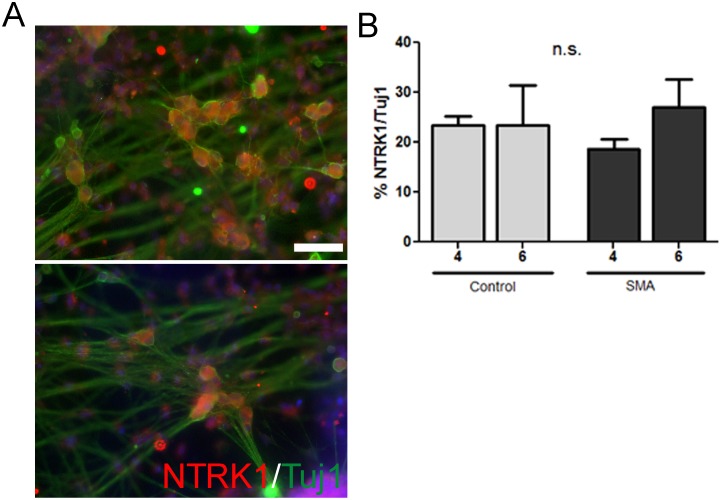
Generation of nociceptive neurons from control and SMA iPSCs. (A) Control and SMA iPSCs generate nociceptive neurons as indicated by NTRK1 (red) and Tuj1 (green). Nuclei are labeled with Hoechst nuclear dye (blue). (B) There was no difference in neuron differentiation between control and SMA iPSC cultures at either 4 or 6 weeks of differentiation. n.s. = not significant by ANOVA. Scale bar = 50 µm.

**Figure 3:**
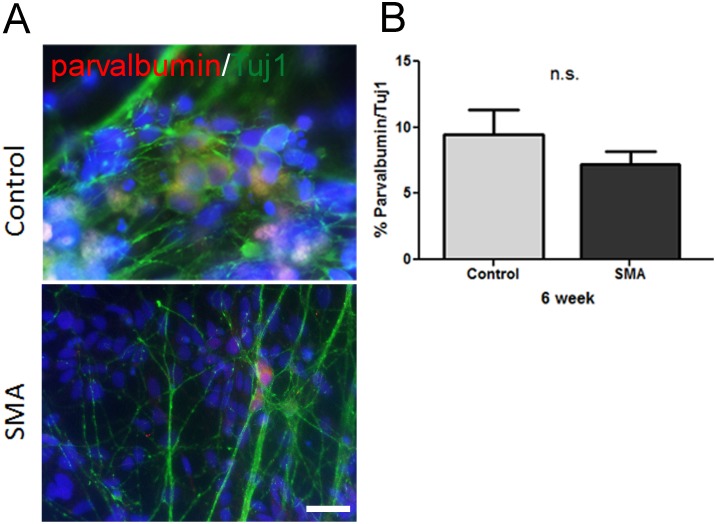
Generation of proprioceptive neurons from control and SMA iPSCs at 6 weeks of differentiation. (A) Both control and SMA iPSCs generate proprioceptive neurons as indicated by parvalbumin (red) and Tuj1 (green). Nuclei are labeled with Hoechst nuclear dye (blue). (B) There was no difference in parvalbumin+neuron number between control and SMA iPSC cultures. n.s. = not significant by t-test. Scale bar = 20 µm.

Peripherin-positive sensory neurons consist of multiple subtypes serving distinct sensory functions such as proprioceptors for limb position and nociceptors for recognizing noxious (i.e. painful) stimuli. We therefore aimed to delineate the subtypes present within our cultures. We first assessed the differentiations for the presence of nociceptors using immunostaining for neurotrophic tyrosine kinase receptor type 1 (NTRK1), also known as TrkA (Figure 2A). We found approximately 20% of the Tuj1 positive neurons in both SMA and control iPSC cultures expressed NTRK1 (Figure 2B). Since previous studies have shown proprioceptive sensory neurons to be affected in SMA [12], [14], we examined whether any proprioceptors were present following differentiation. We used immunostaining for parvalbumin, a marker for large type I proprioceptive sensory neurons and found approximately 5–10% of the neuronal population were parvalbumin positive (Figure 3A, B). Importantly, these data show that unaffected control and SMA iPSC-derived sensory neurons are capable of generating similar numbers of nociceptive and proprioceptive sensory neurons with no significant SMA iPSC-derived sensory neuron loss over time compared to control, regardless of sensory neuron subtype (Figures 1–3). It should be noted that the sensory neuron differentiation in our hands was not as efficient as previously described by others [37]. This could be due to inherent variability of iPSCs, but because all control and SMA iPSC lines used here generated consistent sensory neuron populations, this seems unlikely. However, another possible reason for lowered differentiation efficiency could be due to differences in quantification techniques used; differentiation percentages were based on immunocytochemistry results in unsorted cell cultures rather than by FACS as done by Chambers et al [37]. Nevertheless, our data show no difference between control and SMA iPSCs in regards to sensory neuron differentiation.

**Figure 4:**
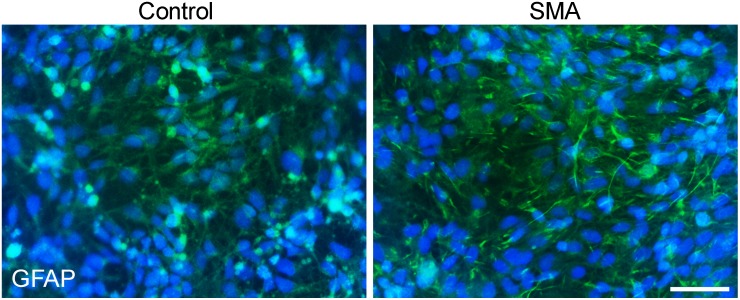
Peripheral glial cells are present in the sensory neuron cultures. GFAP+ glial cells (green) are simultaneously generated during the sensory neuron differentiation in both control and SMA iPSC cultures. Nuclei are labeled with Hoechst nuclear dye (blue). Scale bar = 50 µm.

Because the differentiation protocol generates a mixed population of cells, it is not fully clear what other cellular subtypes are present in the cultures. However, we did analyze the differentiations for the presence of glial cells using GFAP as a marker of Schwann cells, which revealed abundant GFAP+ cells dispersed throughout the culture (Figure 4). Although we cannot completely rule out these are GFAP+ astrocytes, the differentiation protocol proceeds through a Sox10+ neural crest developmental stage (Figure S1 and [37]), so these are more likely to be Schwann cells. We have previously shown that SMA iPSC-derived astrocytes exhibit an activated morphology and show defects in calcium signaling and growth factor expression compared to control iPSC-derived astrocytes [16], which may directly contribute to motor neuron loss. SMA mouse Schwann cells have shown significant myelination defects [40], which necessitates further investigation into iPSC-derived Schwann cell function. However, the stable iPSC-derived sensory neuron cultures over time suggests that functional impairments in SMA iPSC-derived Schwann cells may be minimal or that SMA iPSC-derived sensory neurons are more resistant than motor neurons to glial-induced damage.

### SMA iPSC-derived sensory neurons do not induce motor neuron loss

**Figure 5:**
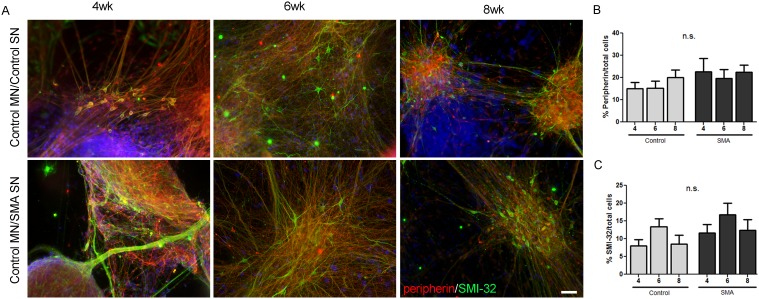
The presence of SMA iPSC-derived sensory neurons does not induce motor neuron loss at 4, 6, and 8 weeks of differentiation. (A) Co-culture of control iPSC-derived SMI-32+ (green) motor neurons (MN) with either SMA iPSC-derived peripherin+ (red) sensory neurons (SN) or control iPSC-derived peripherin+ (red) SNs does not induce motor neuron loss. Nuclei are labeled with Hoechst nuclear dye (blue). Quantification is shown in B and C. n.s. = not significant by ANOVA. Scale bar = 50 µm.

Data from SMA animal models suggest a potential role for sensory neurons in motor neuron demise [12], [14], [15], [26]; therefore, we used direct co-culture methods to test whether SMA iPSC-derived sensory neurons cause loss of healthy control iPSC-derived motor neurons. We followed established differentiation protocols to generate motor neurons from control iPSC lines well characterized in our lab [16], [19], [20], [39]. Control iPSCs were patterned toward motor neurons for 2 weeks followed by direct plating onto 2 week differentiated sensory neuron cultures derived from control and SMA iPSCs. Once plated, the cells were cultured together for an additional 2–6 weeks to allow for maturation. Using immunostaining at 4, 6, and 8 weeks of total differentiation (i.e. 2, 4, and 6 weeks of co-culture), we assessed the numbers of motor neurons and sensory neurons in co-culture utilizing the motor neuron marker SMI-32 and the peripheral sensory neuron marker peripherin. Consistent with being cultured alone (Figure 1), control and SMA iPSC cultures showed similar numbers of sensory neurons in the presence of control motor neurons (Figure 5A, B). Moreover, control motor neurons revealed no statistical difference in cell survival when cultured in the presence of either control or SMA iPSC-derived sensory neurons (Figure 5C). Although a significant reduction in calcium response to KCl induced depolarization was found in SMA iPSC-derived sensory neurons (Figure 1E), this does not appear to negatively impact motor neuron survival. The sensory neuron differentiation medium contains NGF as well as higher concentrations of GDNF and BDNF than what is typically found in the motor neuron differentiation medium. Culture of SMA iPSC-derived motor neurons alone in the sensory neuron differentiation medium does not rescue motor neuron death (Figure S2). Therefore, the absence of a motor neuron phenotype in control motor neuron/SMA sensory neuron iPSC co-cultures is likely not due to culture medium supplementation.

### SMA iPSC-derived sensory neurons do not affect VGLut1 abundance

**Figure 6:**
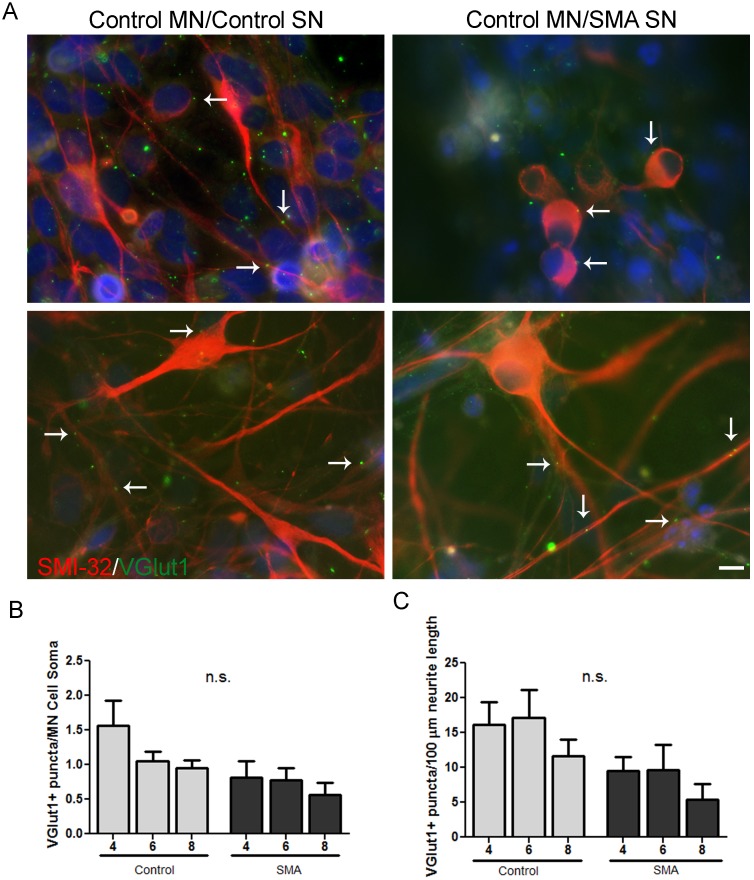
The presence of SMA iPSC-derived sensory neurons (SN) does not induce loss of afferent innervation on control iPSC-derived motor neurons (MN) over 4–8 weeks in culture. (A) VGlut1+ puncta (green) can be identified on both the cell soma and neurites of SMI-32+ control iPSC-derived MNs (red), as indicated by white arrows regardless of being co-cultured with control or SMA iPSC-derived SNs. Nuclei are labeled with Hoechst nuclear dye (blue). There is a trend toward reduced VGlut1+ puncta on both the cell soma (B) and neurites (C) of control iPSC-derived MNs in the presence of SMA iPSC-derived SNs at 4, 6, and 8 weeks in co-culture, but this trend did not reach significance. n.s. = not significant by ANOVA or by Student’s t-test for each time point. Scale bar = 20µm.

Previous work has shown reduced VGlut1+ boutons on the dendrites of SMA motor neurons [10], [12], [14], [25]; therefore, we sought to determine if this was also the case in the SMA/control iPSC co-culture system. To test whether there was a global decrease in VGlut1, we immunostained SMA sensory neuron/control motor neuron co-cultures with SMI-32 and VGlut1 and examined the number of VGlut1+ puncta on both the neurites and cell bodies of SMI-32+ motor neurons (Figure 6A). Quantification of VGlut1 resulted in a trend toward fewer VGlut1+ puncta on control iPSC-derived motor neurons cultured in the presence of SMA iPSC-derived sensory neurons over 6–8 weeks in culture; however, this trend was not statistically different from co-cultures of control iPSC-derived sensory neurons with control iPSC-derived motor neurons (Figure 6B, C). It is possible that after longer times in co-culture the reduction in VGlut1+ boutons would reach significance. However, culturing differentiated iPSC for extended periods of time can lead to sub-optimal culture conditions which may compromise neuronal health and complicate data interpretations. SMA iPSC-derived motor neurons are significantly reduced after 6 weeks in culture [19], [20], but we did not test here whether the addition of SMA iPSC-derived sensory neurons would exacerbate this phenotype. Instead, we tested whether VGlut1 synaptic loss on motor neurons occurs in the absence of sensory neuron co-culture. At 4 weeks of motor neuron differentiation we found that VGlut1+ puncta are significantly reduced on both the neurites and cell bodies of SMA iPSC-derived motor neurons compared to control (Figure S3A, B). These data are consistent with the idea that synapse loss precedes motor neuron death [14], but the loss of VGlut1+ puncta is precipitated by the motor neuron and not the sensory neuron. Taken together, our data suggest that SMA iPSC-derived sensory neurons have a negligible effect on motor neuron afferent innervation and are not sufficient to induce motor neuron loss in this iPSC system.

## Conclusion

Using a human iPSC system, we show that SMA and control iPSCs can be differentiated at equivalent levels into peripheral sensory neurons containing markers of both nociceptors and proprioceptors. As opposed to SMA iPSC-derived motor neurons that die around 6 weeks in culture [19], [20], there is no reduction in neurite length or loss of SMA iPSC-derived sensory neurons over time, which is consistent with the more selective motor neuron loss observed in mouse models and human SMA patients. Moreover, neither the presence of SMA iPSC-derived sensory neurons nor the slight reduction in VGlut1+ bouton numbers on healthy motor neurons is sufficient to induce motor neuron loss. Together with the evidence of VGlut1+ bouton loss on SMA iPSC-derived motor neurons in the absence of sensory neurons, these results suggest that degeneration of the sensory-motor system is likely caused by motor neuron deficits rather than sensory neuron deficits.

## Supporting Information

Figure S1 GFAP+ glial cells (green) produced during the differentiation process express Sox10 (red) indicating these are likely Schwann cells. Images in A and B are taken from two different SMA iPSC lines. Nuclei are labeled with Hoechst nuclear dye (blue). Scale bar = 50 µm.
(TIF)

Figure S2 Culturing SMA iPSC-derived motor neurons in sensory neuron medium does not prevent motor neuron loss. The average number of SMI-32+/Tuj1+ motor neurons at 6 weeks of differentiation was significantly reduced in SMA iPSC cultures maintained in either the standard motor neuron (MN) medium or in the sensory neuron (SN) medium compared to control iPSCs. There was no difference between the two growth conditions for the SMA iPSC cultures. **p = 0.0051 by ANOVA. n.s = not significant by ANOVA.
(TIF)

Figure S3 VGlut1+ puncta are reduced on SMA iPSC-derived motor neurons in the absence of sensory neuron innervation. Four week differentiated SMA iPSC-derived motor neurons exhibit significantly fewer VGlut1+ puncta on both the cell soma (A) and neurites (B) compared to control iPSC-derived motor neurons. *p = 0.0267 by Student’s t-test; **p = 0.0032 by Student’s t-test.
(TIF)

## References

[1] Rochette CF, Gilbert N, Simard LR (2001) SMN gene duplication and the emergence of the SMN2 gene occurred in distinct hominids: SMN2 is unique to Homo sapiens. Hum.Genet. 108: 255–266. 10.1007/s004390100473 11354640

[2] Fischer U, Liu Q, Dreyfuss G (1997) The SMN-SIP1 complex has an essential role in spliceosomal snRNP biogenesis. Cell 90: 1023–1029. 9323130 10.1016/s0092-8674(00)80368-2

[3] Liu Q, Fischer U, Wang F, Dreyfuss G (1997) The spinal muscular atrophy disease gene product, SMN, and its associated protein SIP1 are in a complex with spliceosomal snRNP proteins. Cell 90: 1013–1021. 9323129 10.1016/s0092-8674(00)80367-0

[4] Pellizzoni L, Yong J, Dreyfuss G (2002) Essential role for the SMN complex in the specificity of snRNP assembly. Science 298: 1775–1779. 12459587 10.1126/science.1074962

[5] Lorson CL, Androphy EJ (1998) The domain encoded by exon 2 of the survival motor neuron protein mediates nucleic acid binding. Hum.Mol.Genet. 7: 1269–1275. 10.1093/hmg/7.8.1269 9668169

[6] Lorson CL, Hahnen E, Androphy EJ, Wirth B (1999) A single nucleotide in the SMN gene regulates splicing and is responsible for spinal muscular atrophy. Proc.Natl.Acad.Sci.U.S.A 96: 6307–6311. 10339583 10.1073/pnas.96.11.6307 PMC26877

[7] Lorson CL, Strasswimmer J, Yao JM, Baleja JD, Hahnen E, et al (1998) SMN oligomerization defect correlates with spinal muscular atrophy severity. Nat.Genet. 19: 63–66. 10.1038/ng0598-63 9590291

[8] Monani UR, Lorson CL, Parsons DW, Prior TW, Androphy EJ, et al (1999) A single nucleotide difference that alters splicing patterns distinguishes the SMA gene SMN1 from the copy gene SMN2. Hum.Mol.Genet. 8: 1177–1183. 10.1093/hmg/8.7.1177 10369862

[9] Lotti F, Imlach WL, Saieva L, Beck ES, Hao le T, et al (2012) An SMN-dependent U12 splicing event essential for motor circuit function. Cell 151: 440–454. 23063131 10.1016/j.cell.2012.09.012 PMC3474596

[10] Gogliotti RG, Quinlan KA, Barlow CB, Heier CR, Heckman CJ, et al (2012) Motor neuron rescue in spinal muscular atrophy mice demonstrates that sensory-motor defects are a consequence, not a cause, of motor neuron dysfunction. The Journal of neuroscience: the official journal of the Society for Neuroscience 32: 3818–3829. 22423102 10.1523/JNEUROSCI.5775-11.2012 PMC3679185

[11] Jablonka S, Karle K, Sandner B, Andreassi C, von Au K, et al (2006) Distinct and overlapping alterations in motor and sensory neurons in a mouse model of spinal muscular atrophy. Human Molecular Genetics 15: 511–518. 16396995 10.1093/hmg/ddi467

[12] Ling KK, Lin MY, Zingg B, Feng Z, Ko CP (2010) Synaptic defects in the spinal and neuromuscular circuitry in a mouse model of spinal muscular atrophy. PLoS One 5: e15457. 21085654 10.1371/journal.pone.0015457 PMC2978709

[13] Martinez TL, Kong L, Wang X, Osborne MA, Crowder ME, et al (2012) Survival motor neuron protein in motor neurons determines synaptic integrity in spinal muscular atrophy. The Journal of neuroscience: the official journal of the Society for Neuroscience 32: 8703–8715. 22723710 10.1523/JNEUROSCI.0204-12.2012 PMC3462658

[14] Mentis GZ, Blivis D, Liu W, Drobac E, Crowder ME, et al (2011) Early functional impairment of sensory-motor connectivity in a mouse model of spinal muscular atrophy. Neuron 69: 453–467. 21315257 10.1016/j.neuron.2010.12.032 PMC3044334

[15] Imlach WL, Beck ES, Choi BJ, Lotti F, Pellizzoni L, et al (2012) SMN is required for sensory-motor circuit function in Drosophila. Cell 151: 427–439. 23063130 10.1016/j.cell.2012.09.011 PMC3475188

[16] McGivern JV, Patitucci TN, Nord JA, Barabas ME, Stucky CL, et al (2013) Spinal muscular atrophy astrocytes exhibit abnormal calcium regulation and reduced growth factor production. Glia 61: 1418–1428. 23839956 10.1002/glia.22522 PMC3941074

[17] Voigt T, Meyer K, Baum O, Schumperli D (2010) Ultrastructural changes in diaphragm neuromuscular junctions in a severe mouse model for Spinal Muscular Atrophy and their prevention by bifunctional U7 snRNA correcting SMN2 splicing. Neuromuscular disorders: NMD 20: 744–752. 20832308 10.1016/j.nmd.2010.06.010

[18] Murray LM, Beauvais A, Bhanot K, Kothary R (2012) Defects in neuromuscular junction remodelling in the Smn(2B/−) mouse model of spinal muscular atrophy. Neurobiology of Disease 49C: 57–67. 10.1016/j.nbd.2012.08.019 22960106

[19] Ebert AD, Yu J, Rose FF Jr, Mattis VB, Lorson CL, et al (2009) Induced pluripotent stem cells from a spinal muscular atrophy patient. Nature 457: 277–280. 19098894 10.1038/nature07677 PMC2659408

[20] Sareen D, Ebert AD, Heins BM, McGivern JV, Ornelas L, et al (2012) Inhibition of apoptosis blocks human motor neuron cell death in a stem cell model of spinal muscular atrophy. PLoS One 7: e39113. 22723941 10.1371/journal.pone.0039113 PMC3378532

[21] Anagnostou E, Miller SP, Guiot MC, Karpati G, Simard L, et al (2005) Type I spinal muscular atrophy can mimic sensory-motor axonal neuropathy. Journal of child neurology 20: 147–150. 15794183 10.1177/08830738050200022101

[22] Omran H, Ketelsen UP, Heinen F, Sauer M, Rudnik-Schoneborn S, et al (1998) Axonal neuropathy and predominance of type II myofibers in infantile spinal muscular atrophy. Journal of child neurology 13: 327–331. 9701481 10.1177/088307389801300704

[23] Rudnik-Schoneborn S, Goebel HH, Schlote W, Molaian S, Omran H, et al (2003) Classical infantile spinal muscular atrophy with SMN deficiency causes sensory neuronopathy. Neurology 60: 983–987. 12654964 10.1212/01.wnl.0000052788.39340.45

[24] Thirumalai V, Behrend RM, Birineni S, Liu W, Blivis D, et al (2013) Preservation of VGLUT1 synapses on ventral calbindin-immunoreactive interneurons and normal locomotor function in a mouse model of spinal muscular atrophy. Journal of Neurophysiology 109: 702–710. 23136344 10.1152/jn.00601.2012 PMC3567388

[25] Park GH, Maeno-Hikichi Y, Awano T, Landmesser LT, Monani UR (2010) Reduced survival of motor neuron (SMN) protein in motor neuronal progenitors functions cell autonomously to cause spinal muscular atrophy in model mice expressing the human centromeric (SMN2) gene. The Journal of neuroscience: the official journal of the Society for Neuroscience 30: 12005–12019. 20826664 10.1523/JNEUROSCI.2208-10.2010 PMC2944776

[26] Zhang Z, Pinto AM, Wan L, Wang W, Berg MG, et al (2013) Dysregulation of synaptogenesis genes antecedes motor neuron pathology in spinal muscular atrophy. Proceedings of the National Academy of Sciences of the United States of America 110: 19348–19353. 24191055 10.1073/pnas.1319280110 PMC3845193

[27] Hultborn H (2006) Spinal reflexes, mechanisms and concepts: from Eccles to Lundberg and beyond. Progress in Neurobiology 78: 215–232. 16716488 10.1016/j.pneurobio.2006.04.001

[28] Leveille F, Papadia S, Fricker M, Bell KFS, Soriano FX, et al (2010) Suppression of the Intrinsic Apoptosis Pathway by Synaptic Activity. Journal of Neuroscience 30: 2623–2635. 20164347 10.1523/JNEUROSCI.5115-09.2010 PMC2834927

[29] Catsicas M, Pequignot Y, Clarke PG (1992) Rapid onset of neuronal death induced by blockade of either axoplasmic transport or action potentials in afferent fibers during brain development. The Journal of neuroscience: the official journal of the Society for Neuroscience 12: 4642–4650. 1281491 10.1523/JNEUROSCI.12-12-04642.1992 PMC6575767

[30] Ikonomidou C, Bosch F, Miksa M, Bittigau P, Vockler J, et al (1999) Blockade of NMDA receptors and apoptotic neurodegeneration in the developing brain. Science 283: 70–74. 9872743 10.1126/science.283.5398.70

[31] Tsai LK, Tsai MS, Ting CH, Wang SH, Li H (2008) Restoring Bcl-x(L) levels benefits a mouse model of spinal muscular atrophy. Neurobiology of Disease 31: 361–367. 18590823 10.1016/j.nbd.2008.05.014

[32] Tsai MS, Chiu YT, Wang SH, Hsieh-Li HM, Lian WC, et al (2006) Abolishing Bax-dependent apoptosis shows beneficial effects on spinal muscular atrophy model mice. Molecular therapy: the journal of the American Society of Gene Therapy 13: 1149–1155. 16564230 10.1016/j.ymthe.2006.02.008

[33] Kerr DA, Nery JP, Traystman RJ, Chau BN, Hardwick JM (2000) Survival motor neuron protein modulates neuron-specific apoptosis. Proceedings of the National Academy of Sciences of the United States of America 97: 13312–13317. 11078511 10.1073/pnas.230364197 PMC27221

[34] Takahashi K, Tanabe K, Ohnuki M, Narita M, Ichisaka T, et al (2007) Induction of pluripotent stem cells from adult human fibroblasts by defined factors. Cell 131: 861–872. 18035408 10.1016/j.cell.2007.11.019

[35] Yu J, Hu K, Smuga-Otto K, Tian S, Stewart R, et al (2009) Human induced pluripotent stem cells free of vector and transgene sequences. Science 324: 797–801. 19325077 10.1126/science.1172482 PMC2758053

[36] Yu J, Vodyanik MA, Smuga-Otto K, Antosiewicz-Bourget J, Frane JL, et al (2007) Induced pluripotent stem cell lines derived from human somatic cells. Science 318: 1917–1920. 18029452 10.1126/science.1151526

[37] Chambers SM, Qi Y, Mica Y, Lee G, Zhang XJ, et al (2012) Combined small-molecule inhibition accelerates developmental timing and converts human pluripotent stem cells into nociceptors. Nature Biotechnology 30: 715–720. 10.1038/nbt.2249 PMC3516136 22750882

[38] HD iPSC Consortium (2012) Induced Pluripotent Stem Cells from Patients with Huntington’s Disease Show CAG Repeat Expansion-Associated Phenotypes. Cell Stem Cell 11: 264–278. 22748968 10.1016/j.stem.2012.04.027 PMC3804072

[39] Ebert AD, Shelley BC, Hurley AM, Onorati M, Castiglioni V, et al (2013) EZ spheres: A stable and expandable culture system for the generation of pre-rosette multipotent stem cells from human ESCs and iPSCs. Stem Cell Research 10: 417–427. 23474892 10.1016/j.scr.2013.01.009 PMC3786426

[40] Hunter G, Aghamaleky Sarvestany A, Roche SL, Symes RC, Gillingwater TH (2014) SMN-dependent intrinsic defects in Schwann cells in mouse models of spinal muscular atrophy. Human Molecular Genetics 23: 2235–2250. 24301677 10.1093/hmg/ddt612

