# Supplementary figures and images for: Sensory Neurons Do Not Induce Motor Neuron Loss in a Human Stem Cell Model of Spinal Muscular Atrophy
Source: PLoS One. 2014 Jul 23;9(7):e103112. doi: 10.1371/journal.pone.0103112 (PMC4108398; doi:10.1371/journal.pone.0103112)

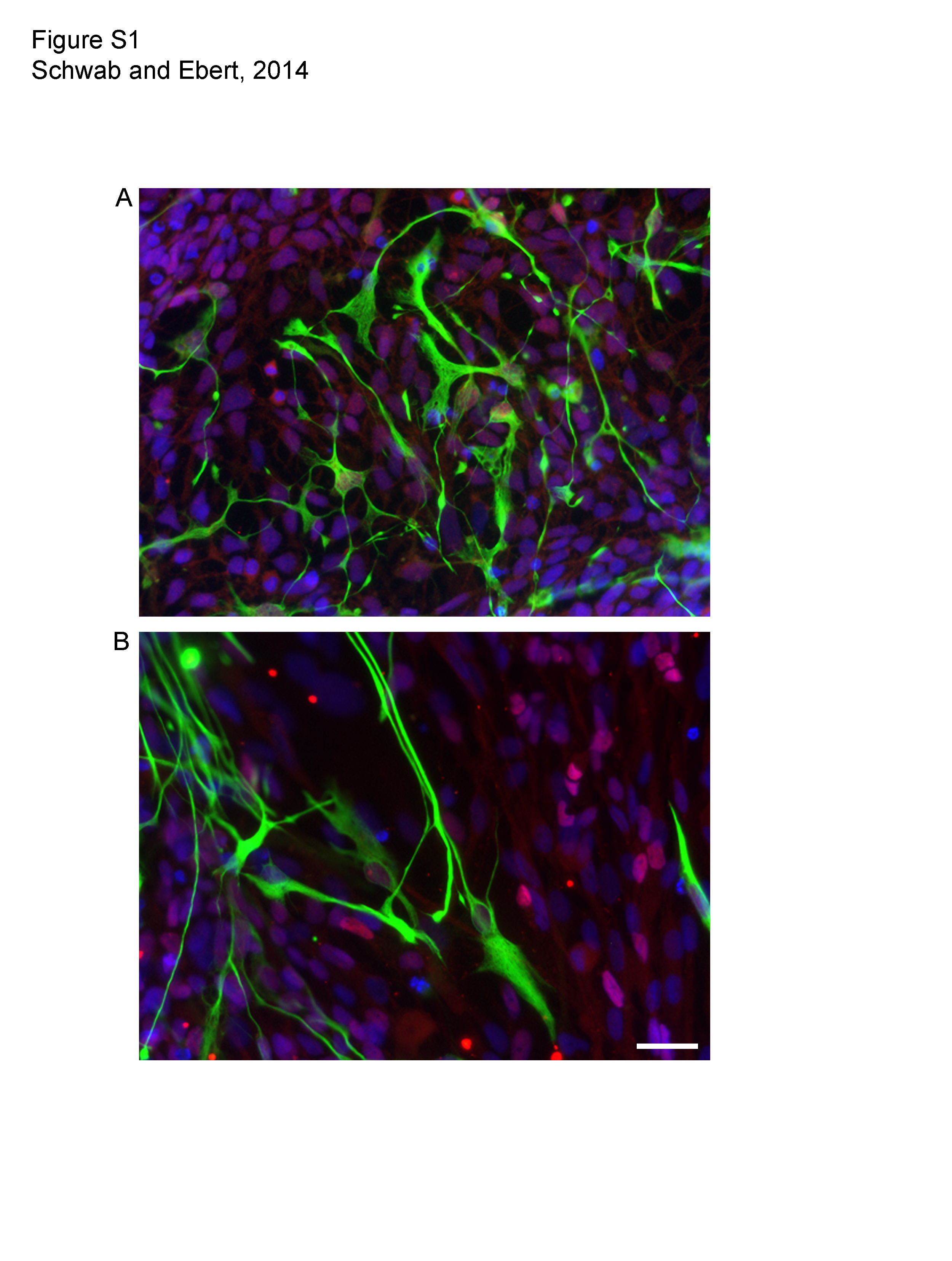

Supplement: Figure S1 — GFAP+ glial cells (green) produced during the differentiation process express Sox10 (red) indicating these are likely Schwann cells. Images in A and B are taken from two different SMA iPSC lines. Nuclei are labeled with Hoechst nuclear dye (blue). Scale bar = 50 µm. (TIF) [file pone.0103112.s001.tif]

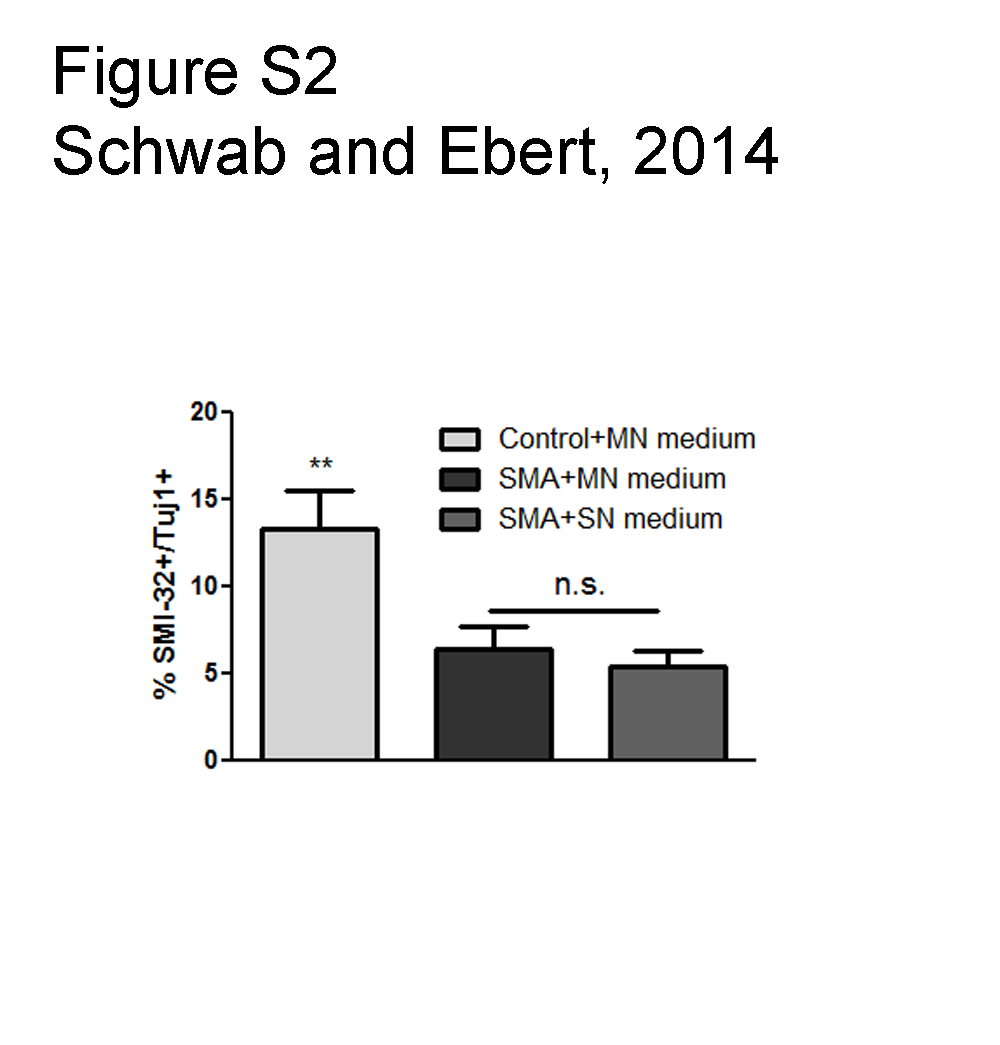

Supplement: Figure S2 — Culturing SMA iPSC-derived motor neurons in sensory neuron medium does not prevent motor neuron loss. The average number of SMI-32+/Tuj1+ motor neurons at 6 weeks of differentiation was significantly reduced in SMA iPSC cultures maintained in either the standard motor neuron (MN) medium or in the sensory neuron (SN) medium compared to control iPSCs. There was no difference between the two growth conditions for the SMA iPSC cultures. **p = 0.0051 by ANOVA. n.s = not significant by ANOVA. (TIF) [file pone.0103112.s002.tif]

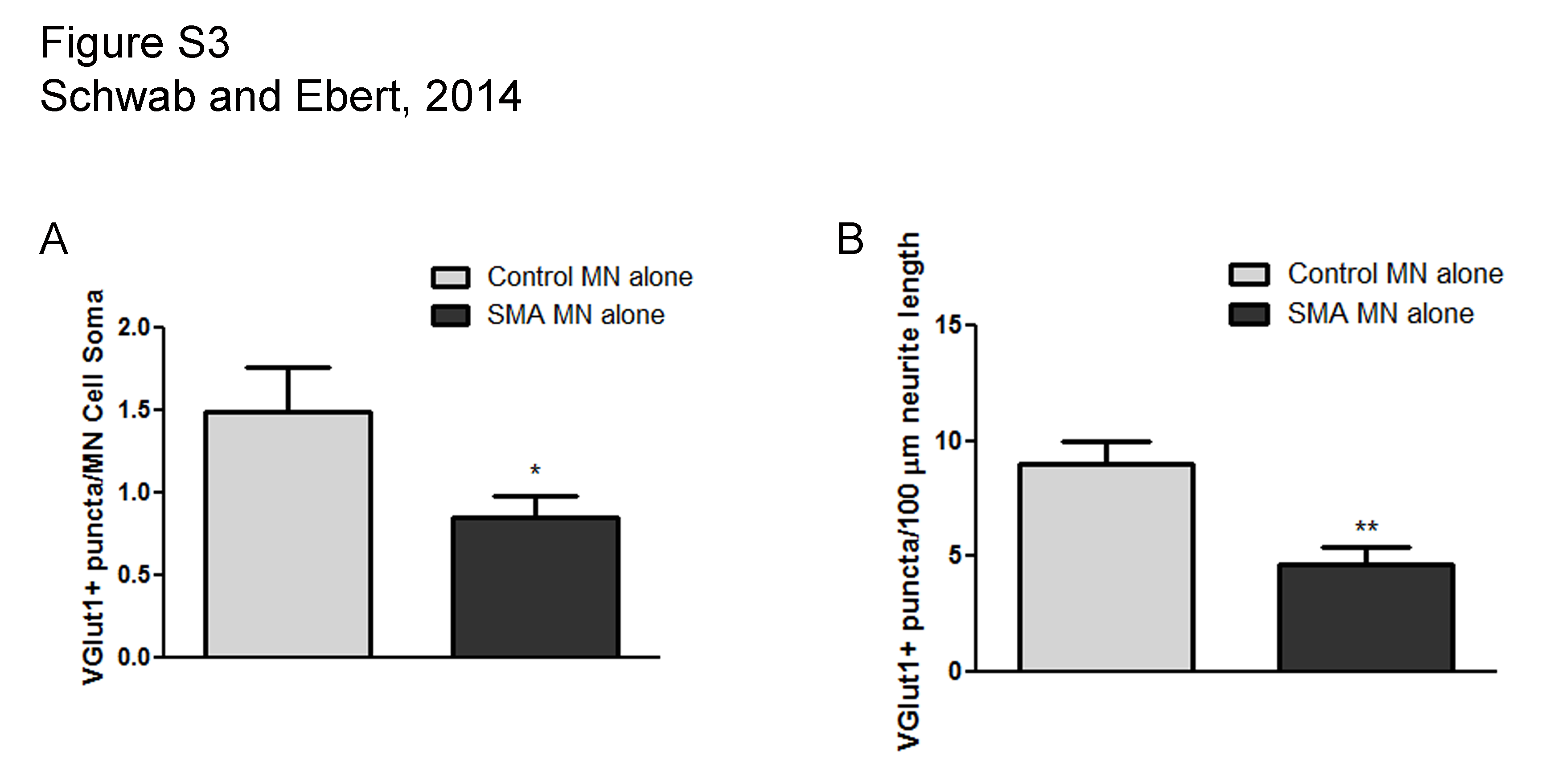

Supplement: Figure S3 — VGlut1+ puncta are reduced on SMA iPSC-derived motor neurons in the absence of sensory neuron innervation. Four week differentiated SMA iPSC-derived motor neurons exhibit significantly fewer VGlut1+ puncta on both the cell soma (A) and neurites (B) compared to control iPSC-derived motor neurons. *p = 0.0267 by Student’s t-test; **p = 0.0032 by Student’s t-test. (TIF) [file pone.0103112.s003.tif]
